# Innovative Textiles Used in Face Masks: Filtration Efficiency and Self-Disinfecting Properties against Coronaviruses

**DOI:** 10.3390/nano11082088

**Published:** 2021-08-17

**Authors:** Paul Siller, Janina Reissner, Sabrina Hansen, Michael Kühl, Alexander Bartel, David Schmelzeisen, Thomas Gries, Uwe Roesler, Anika Friese

**Affiliations:** 1 Institute for Animal Hygiene and Environmental Health, Freie Universitaet Berlin, 14163 Berlin, Germany; paul.siller@fu-berlin.de (P.S.); janina.reissner@fu-berlin.de (J.R.); sabrina.hansen@fu-berlin.de (S.H.); michael.kuehl@fu-berlin.de (M.K.); Uwe.Roesler@fu-berlin.de (U.R.); 2 Institute for Veterinary Epidemiology and Biostatistics, Freie Universitaet Berlin, 14163 Berlin, Germany; alexander.bartel@fu-berlin.de; 3 Institut für Textiltechnik, RWTH Aachen University, 52062 Aachen, Germany; david.schmelzeisen@ita.rwth-aachen.de (D.S.); thomas.gries@ita.rwth-aachen.de (T.G.)

**Keywords:** textile, nanofleece, coronavirus, SARS-CoV-2, face mask, antiviral, aerosol

## Abstract

Within the current SARS-CoV-2 pandemic, personal protective equipment, including face masks, is one important tool to interrupt virus transmission chains within the community. In this context, the quality of different face masks is frequently discussed and should, therefore, be evaluated. In this study, nanofleece textiles with a particle filtering effect and textiles with a self-disinfecting treatment were examined, which may be combined in face masks. Firstly, newly developed nanofleece textiles were tested regarding their filtration efficiency against airborne coronavirus, using feline coronavirus (FCoV) as a surrogate for SARS-CoV-2. The tested nanofleece textiles showed filtration efficiencies of over 95% against FCoV when used as a double layer and were, therefore, almost on par with the FFP-2 mask material, which was used as a reference. Secondly, eight treated, self-disinfecting textiles, which may increase the safety in the handling of potentially contaminated masks, were tested against SARS-CoV-2. Three out of eight treated textiles showed significant activity against SARS-CoV-2 and achieved about three LOG_10_ (99.9%) of virus titer reduction after twelve hours of incubation. Since all possible transmission paths of SARS-CoV-2, as well as the minimal infection doses, remain unknown, both investigated approaches seem to be useful tools to lower the virus spread within the community.

## 1. Introduction

As a result of the spread of the severe acute respiratory syndrome coronavirus 2 (SARS-CoV-2), we are experiencing a pandemic with far-reaching effects on social life and the economy worldwide [1]. According to current data (9 August 2021) from John Hopkins University, over 202 million people were infected and over 4.2 million people have died as a result of coronavirus disease-2019 (COVID-19), the disease caused by SARS-CoV-2 (https://coronavirus.jhu.edu/map.html, accessed on 9 August 2021). In an attempt to control the spread of the virus, protective measures are being taken worldwide. The World Health Organization recommends increased hand hygiene by disinfection with alcohol-based disinfectants and frequent handwashing, keeping a distance from other people (>1 m) and wearing face masks. It is known that face masks retain droplets, prevent the generation of virus aerosols by the wearer and, therefore, protect others and also probably the wearer [2]. At the beginning of the pandemic, droplets were assumed to be the main transmission path of SARS-CoV-2. There is now, however, evidence to suggest that transmission via aerosols plays a key role in the pandemic [3]. In particular, the very small, airborne droplets containing the virus, which can develop very rapidly from larger droplets after exhalation, do not sink but remain in the air and transport the virus [4]. Face masks were shown to significantly reduce the airborne spread of various respiratory pathogens [5] including SARS-CoV-2 [6]. The term face mask is used synonymously with personal protective equipment, including respirators, for the general population in the ongoing pandemic. Face masks and respirators, which offer different levels of protection to users, are available. In general, respirators fit tightly compared to face masks. Face masks may also be classified according to their reusability. Commonly used reusable masks include homemade and commercial cloth masks, while disposable ones include surgical masks, N95 and KN95 respirators [7].

There has been a scarcity of effective personal protective equipment (PPE) such as N95 masks since the beginning of the pandemic [8], which continues to date. Newly developed, cost-efficient textiles with high filtration efficiencies against coronaviruses could help to avoid bottlenecks in the supply chain, which are mainly occurring in developing countries [9]. Further, N95 masks have limited reusability [10]. The filtration efficiency of N95 masks is dependent on the electrostatic effect of an intermediate layer of charged polypropylene. This charge can degrade by using or decontaminating the mask [11]. N95 respirators should, therefore, be changed frequently, as the electrostatic filtration efficiency will significantly decrease when the respirator becomes moist, e.g., through exhalation [12]. Masks with improved mechanical filter technologies, such as electrospun nonwovens, could pose a promising alternative. Electrospun nanofibers have excellent air filtering properties including a large surface-to-volume ratio properties, a good interconnectivity and a micrometer-sized interstitial space [13]. Additionally, electrospun nanomaterials have a stable residual charge [14], which is suspected to further increase the filtration efficiency regarding aerosolized particles [15]. Furthermore, electro spun nanofilters exhibit superior breathing comfort with good thermal behavior based on the transmission properties for air, moisture and carbon dioxide [16].

In addition to airborne spread, contaminated objects and surfaces may also contribute to the spread of the virus. Several studies showed that SARS-CoV-2 can remain infectious on various surfaces and textiles at room temperature for between three [17] and seven days (outer layer of surgical mask) [18]. The possibility of spreading respiratory viruses via contaminated, dry surfaces is considered likely, at least in hospital settings [19]. This route has already been proven for enteric viruses [20]. Besides aerosol transmission, these smear infections of SARS-CoV-2 may be prevented by self-disinfecting textile surfaces [21]. Surfaces of protective clothing, such as face masks, but also everyday objects such as clothing or furniture and the interiors of cars, trains, or airplanes, are mainly covered by textiles. To equip textiles with antibacterial and antiviral properties, textile fibers can be made from materials with antibacterial and antiviral properties (such as copper or platinum) or they can be treated with antimicrobial substances.

The focus of the second part of this study was the investigation of the antiviral activity of textile treatments against SARS-CoV-2. The investigated treatments can be applied in the finishing step of textile production. This allows high flexibility and fast reactions to arising needs. When selecting the finishes, substances based on heavy metals were excluded since discussion of their effects on health and the environment has, so far, been met with controversy [22].

The overall aim of this study was to evaluate newly developed textiles used in PPE regarding their filtration efficiency and self-disinfecting effect against Coronaviruses.

## 2. Materials and Methods

In the context of this study, two types of textiles are to be distinguished according to their intended use: For the first part of the study, where the aim was the filtering of virus-containing air particles in the range of a few micrometers, voluminous melt-spun (meltblown) and commercially available electrospun (nano) nonwoven materials, optimized for maximum airflow and high filtration efficiency, were used. For the second part of the study, in which textiles were treated with antiviral substances, the textiles had to fulfill other criteria, mainly filter protection, moisture regulation, wearer comfort and hygiene. Therefore, in this part of the study, woven and knitted fabrics made of the most widely used materials by worldwide production quantity in metric tons in 2020, polyester (52.2%) and cotton (23.2%) [23], were examined.

### 2.1. Filtration Efficiency Trials

#### 2.1.1. Characterization of Mask Materials

**Figure 1:**
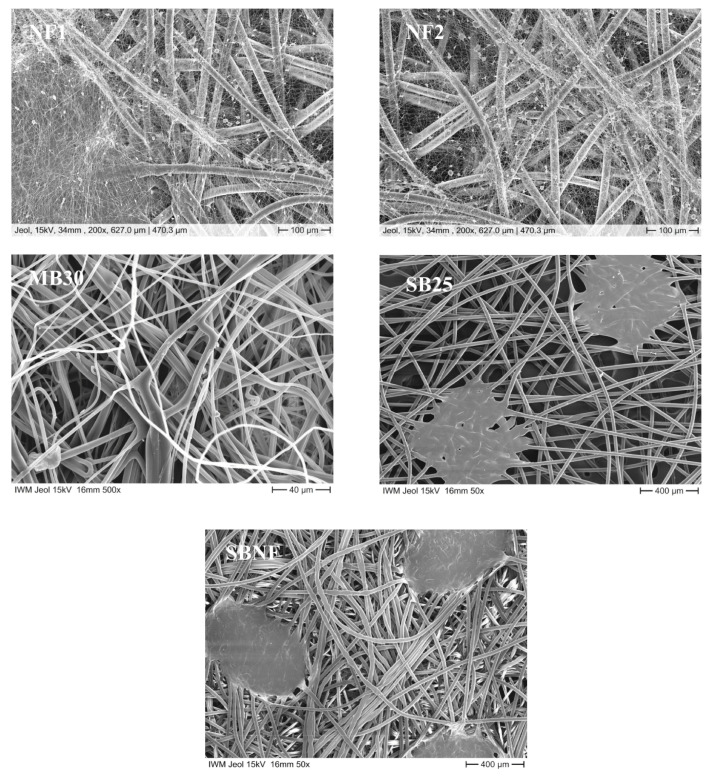
Microscopic images of nonwoven textile samples. Nanofleece 1 (NF1) and nanofleece 2 (NF2) layers were imaged at a magnification of 200×. The meltblown textile was imaged at a magnification of 500×. The carrier spunbond layers of the Meltblown (SB25) and the Nanofleeces (SBNF) were imaged at a magnification of 50×.

The characterization of the nonwovens used is based on the manufacturing process, the polymer used, their weight per unit area (DIN EN 29,073 Part 1), their thickness (DIN EN ISO 9073-2) and their air permeability (DIN EN ISO 9073 Part 15). Details regarding the properties of the nonwoven materials used in this study are depicted in Table S1 in the supplementary materials. For material NF2, the thickness could not be determined, because it was below the measurement range of the thickness measurement device (No. 16,052, Frank PTI GmbH, Birkenau, Germany). Microscopic images showing the structure of the nonwoven textile samples are displayed in Figure 1.

Masks available on the market differ greatly in their design. The EN149 standard, in which the requirements for masks of class FFP-2 (filtering face piece-2) are standardized, only specifies minimum values for the different test categories. The required filtering efficiency of the FFP-2 European standard is comparable to the N95 (USA) and KN95 standard (China) for crystalline particles such as NaCl.

Seven combinations of the mask materials were investigated regarding their filtration efficiency against airborne FCoV. Two electrospun nonwoven materials were included, which were both tested as a single (NFA, NFB) and double layer (NFA2, NFB2). NFA2 was additionally tested after 30 cycles of washing in a washing machine at 60 °C according to DIN EN ISO 6330 (NFA2W). Additionally, three reference mask materials were tested: The layers of the different nanofleece mask materials and all references are described in Table 1. For the community mask material, a canvas woven cotton material was chosen.

For the initial characterization of the materials concerning their leakage for NaCl particles, all combinations were tested in an aerosol chamber at the Leibniz Institute for Interactive Materials (Aachen, Germany). Each textile sample was placed between two plates in the aerosol chamber and had NaCl particles of between 90 and 500 nanometers, generated by a Portable Test Aerosol Generator Model 3073 (TSI GmbH, Aachen, Germany), passed through it. The amount of particles that passed through were measured with the Laser Aerosol Spectrometer Model 3340A (TSI GmbH, Aachen, Germany) behind the filter layer. An initial measurement was made without any textile between the plates to determine a baseline of the NaCl particles passing through without any filtration. The reference measurement with the FFP2 mask material showed a filtration efficiency of 89% of the chosen particle distribution. The results of this NaCL Test are depicted in Table 1. For NFB and NFB2, we suspect a measurement error, because the nanofleece layer showed a detachment from the spunbond layer while measuring. Therefore, these data are not included.

#### 2.1.2. Experimental Setup for the Viral Filtration Efficiency Trials

For testing of the viral filtration efficiency of different mask materials, an FCoV aerosol was passed through the textiles in a miniaturized aerosol chamber and the virus concentration in the air in front of and behind the fabric was determined.

**Figure 2:**
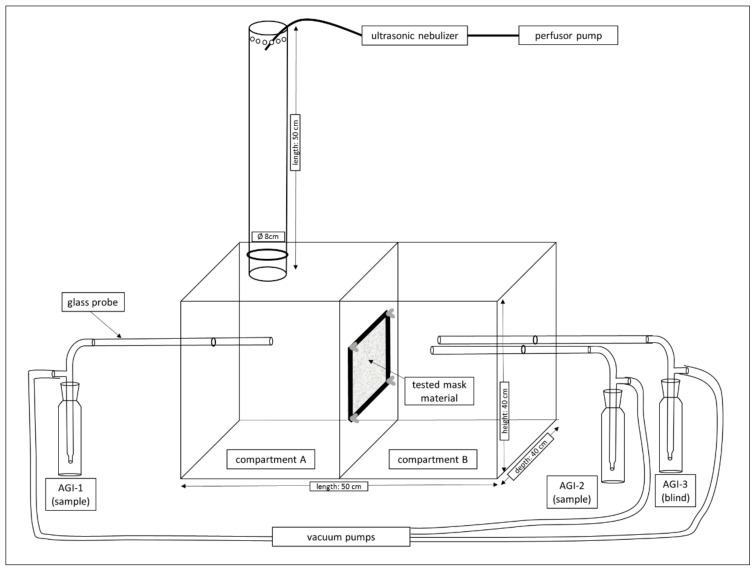
Experimental setup used for the filtration efficiency trials.

The aerosol chamber consisted of acrylic glass with an antistatic coating. It had a length of 50 cm and a height and depth of 40 cm each, resulting in a total volume of 80 L (Figure 2). It was divided into two equal compartments (A and B). In between, the mask material was fixed in a circular cutout with an opening of 50 cm^2^. Air was aspirated from compartment B at a flow rate of 12.5 L/min by connecting an all-glass impinger 30 (AGI-30, Neubert Glas GbR, Geschwenda, Germany, VDI Norm 4252-3) upstream of a vacuum pump (Leybold S4B, Leybold, Cologne, Germany and Edwards RV3, Edwards, Feldkirchen, Germany). The virus suspension was nebulized using an ultrasonic nebulizer (Sono-Tek, Milton, NY, USA) and entered compartment A through a nozzle (Sono-Tek, Milton, NY, USA) from above via an acrylic glass tube. Viral suspensions previously loaded into 50 mL syringes were transported to the ultrasonic nebulizer by a perfusion pump at 36 mL/h. After an equilibration period of 10 min, air samples were simultaneously taken in both compartments of the aerosol chamber with AGI-30 impingers. Subsequently, the concentration of infectious FCoV in the air samples was determined. The aerosol chamber was placed in a hermetically sealed room. Each mask material was tested five times. The particle size distribution generated by the ultrasonic nebulizer in the aerosol chamber was determined with a Lighthouse 3016 handheld device (Lighthouse worldwide solutions, Fremont, CA, USA). This measurement was carried out at a low RH (relative humidity) of approximately 30% because at high RH, the measurements of light-scattering based particle counters are erroneous [24].

#### 2.1.3. Air Sampling

One AGI-30 used for sampling was connected to compartments A and B, respectively. Therefore, glass probes with an internal diameter of 7 mm and a length of 25 cm were used, enabling air sampling in the center of the chamber’s compartments. The impingers were connected to vacuum pumps, resulting in an airflow of 12.5 L/min for each impinger, which was monitored using a rotameter. After the equilibration period, both impingers collected air samples for 15 min in parallel. The impingers were filled with 30 mL of Dulbecco’s Modified Eagle Medium (DMEM High Glucose, Biowest, Nuaillé, France.) with the addition of 1% fetal bovine serum (FBS, PAN Biotech, Aidenbach, Germany). Additionally, 3 mL of a solution containing 10,000 IU/mL Penicillin, 10,000 µg/mL Streptomycin and 25 µg/mL Amphotericin B was added (Biozym, Hessisch Oldendorf, Germany) to the air sampling fluid, as well as 50 µL of autoclaved linseed oil to avoid foam formation.

The temperature and RH were continuously monitored with data loggers (LOG210 5005-0210, Dostmann electronics, Wertheim, Germany) in 20 s intervals in both compartments of the aerosol chamber.

#### 2.1.4. Preparation of Viral Suspensions

For all aerosol experiments, an FCoV strain was used as a surrogate for SARS-CoV2. It was provided by the Friedrich Loeffler Institute (FLI, Isle of Riems, Germany; viral registration number RVB-1259).

The FCoV virus was propagated on Crandell-Rees Feline Kidney (CRFK) cells (ATTC CCL-94; https://www.lgcstandards-atcc.org/Products/All/CCL-94.aspx?geo, accessed on 13 August 2021) in DMEM High Glucose supplemented with 10% fetal bovine serum (PAN Biotech, Aidenbach, Germany), 100 IU/mL penicillin G and 100 µg/mL streptomycin (Biochrom AG, Berlin, Germany). In detail, CRFK cells were grown in T-175 tissue culture flasks (Sarstedt, Nürmbrecht, Germany) and infected with 400 µL of FCoV virus with a titer of approximately 10^5.5^ median tissue culture infectious dose _50_ (TCID_50_)/mL. After a three-day incubation period at 37 °C in a 5% CO_2_ atmosphere, flasks were frozen at −20 °C for at least 24 h. Afterward, the virus-containing cell culture medium in the flasks was thawed and cell detritus was removed by centrifugation. The virus concentration of the supernatant was determined and stored at −80 °C until usage. For aerosolization, the concentration was adjusted to approximately 10^6.225^ TCID_50_/mL for each of the experiments.

#### 2.1.5. Quantification of the Viral Suspensions and Air Samples

Viral suspensions and air samples were quantified by calculating the TCID_50_ using an endpoint dilution assay and calculation according to the Spearman–Karber method [25,26]. For viral titration, serial dilutions of the suspensions and air samples were prepared. Afterward, each dilution was transferred to CRFK cells in 96-well plates (Sarstedt, Nürmbrecht, Germany) in an eightfold approach and incubated at 37 °C in a 5% CO_2_ atmosphere. Each well was checked for a cytopathic effect (CPE) after approximately 60 h, 84 h and 108 h. Air samples were filtered with filters of 0.22 µm pore size (Roth, Karlsruhe, Germany) before titration. 

### 2.2. Textile Surface Treatment Trials

#### 2.2.1. Tested Textiles

Eight treatments based on quaternary ammonium compounds (QAC) of six different textiles were tested for their antiviral activity against SARS-CoV-2. Details about the material composition and treatments are shown in Table 2.

The characterization of the materials is based on the material, the type of production (woven, knitted), their specific weight (DIN EN 12,127), their thickness (DIN EN ISO 5084), their air permeability (DIN EN ISO 9237), the longitudinal and transverse thread density (DIN EN 1049 Part 2) for woven fabrics and the longitudinal and transverse mesh count (DIN EN 14,971) for knitted fabrics. Canvas materials are durable, woven textiles. The numbers in the textile names indicate the specific weight in g/m^2^. The characteristics of the woven and knitted textiles are displayed in Table S2 (Supplementary Materials).

#### 2.2.2. Virus and Cell Lines

The variant of SARS-CoV-2 used was SARS-CoV-2 München (SARS-CoV-2M; [27]). The isolate was handled under the appropriate safety precautions in a BSL-3 facility (Freie Universität Berlin, Department for Veterinary Medicine). The virus used for the testing procedure was propagated on Vero E6 cells (ATCC CRL-1586; https://www.lgcstandards-atcc.org/products/all/crl-1586.aspx, accessed on 13 August 2021) in Minimum Essential Medium–Eagle with Earle’s BSS (Lonza, Basel, Switzerland) supplemented with 10% fetal bovine serum (PAN Biotech, Aidenbach, Germany), 100 IU/mL penicillin G, 100 µg/mL streptomycin (Biochrom AG, Berlin, Germany) and 1% NEA (Biochrom AG, Berlin, Germany). This specific medium does not contain phenol red and HEPES (4-(2-hydroxyethyl)-1-piperazineethanesulfonic acid), a zwitterionic sulfonic acid buffering agent. The medium was chosen to prevent an interaction of treated textiles with the mentioned substances due to electrical charges. Virus titrations were performed in 96-well plates on Vero E6 cell in DMEM High Glucose supplemented with 10% fetal bovine serum and 100 IU/mL penicillin G and 100 µg/mL streptomycin.

#### 2.2.3. Antiviral Activity Test of Textiles

The antiviral activity tests were modified according to ISO 18,184:2014. All textiles were washed with deionized water ten times at 40 °C and dried afterward. The textiles were cut into pieces of approximately 20 × 20 mm and portions with a mass of 0.4 ± 0.02 g were prepared. The textiles were sterilized at 121 °C for 15 min. The day before the test procedure, all textiles were conditioned overnight at 37 °C at high air moisture.

Before starting the test procedure, various control tests were performed according to ISO 18,184: 2014 to confirm that there is no cytotoxic effect, no reduction in cell sensitivity to the virus and a reliable inactivation of antiviral activity. When all controls were verified, the tests were performed as follows.

For a first screening, the antiviral activity of the treated textiles after 6 h of virus suspension application was investigated. Therefore, portions of untreated and treated textile samples were inoculated with 1 mL of virus suspension each in duplicate. For textile N, a 0.5 mL suspension per portion was used since the absorption capacity was too low for 1 mL fluid. The inoculum had a virus titer of 10^7^ TCID_50_ per portion. After adsorption, the textile samples were uniformly moistened. After 6 h incubation at room temperature, 19 mL (or 19.5 mL for textile N) washing solution was added and the samples were vortexed thoroughly. The solution was quantified by calculating the TCID_50_ using an endpoint dilution assay and calculation according to the Spearman–Karber method as described above. The virus titer was compared between treated and untreated samples.

Textiles with an antiviral active treatment were selected and further investigated. The procedure was repeated for additional incubation times (0.5 h, 1 h, 2 h, 6 h and 12 h) and in a four-fold approach. The virus titer was compared between treated and untreated samples for the specific time points.

### 2.3. Statistical Analysis

All statistical analysis was performed using R version 4.04 (R Foundation Vienna; Austria). Figures were created using Graphpad Prism 8 (GraphPad Software, San Diego, CA, USA). For statistical analysis, we used Poisson regression with robust standard errors to account for overdispersion. For the filtration efficiency trials, we used the TCID_50_ virus titer in compartment B as the dependent variable and the TCID_50_ in compartment A as an offset to calculate the relative reduction in virus titer between both compartments. The reduction was calculated separately for each mask material by adding a fixed effect. Post-hoc multiple comparisons between all mask materials were adjusted using the Tukey method. For the antiviral activity trials, the TCID_50_ at the end of the incubation time was used as the dependent variable. The interaction between textile, treatment and incubation time was used as a fixed effect. Estimated marginal means with a 95% confidence interval were calculated for each combination of the 3 parameters. Tukey-adjusted multiple comparisons between all textile and treatment combinations were calculated separately for each timepoint. Model-based estimated marginal means [28] with confidence intervals and multiple comparison post-hoc tests were calculated using the emmeans R package (version 1.5.4; https://cran.r-project.org/web/packages/emmeans/index.html, accessed on 13 August 2021). Robust standard errors were calculated using the sandwich R package (version 3.0-0; https://cran.r-project.org/web/packages/sandwich/index.html, accessed on 13 August 2021 [29]). Results are reported with 95% confidence intervals. A significance threshold of 0.05 was used.

## 3. Results

### 3.1. Filtration Efficiency of the Investigated Mask Materials against Aerosolized FCoV

**Figure 3:**
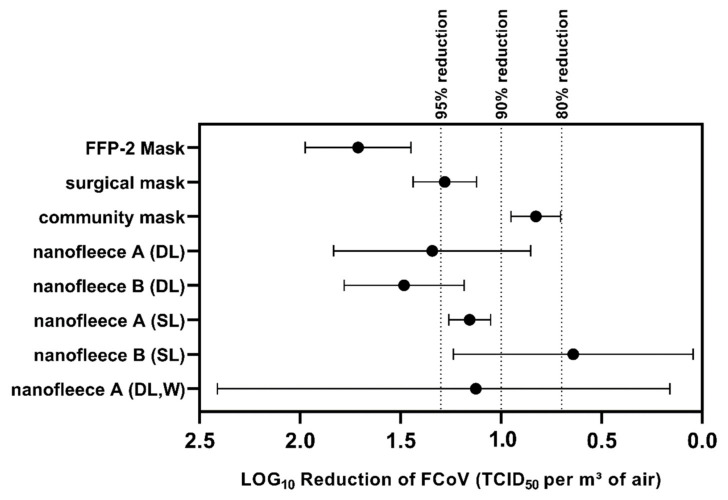
LOG_10_ Reduction of infective FCoV in the aerosol for different tested filter materials in TCID_50_/m^3^. The error bars indicate the 95% confidence intervals.

The data on the filtration efficiency of the tested mask materials are summarized in Figure 3. Concerning the tested reference masks, a mean reduction of 1.71 LOG_10_ TCID_50_ of FCoV per m^3^ of air (98.1%) was observed for the FFP-2 mask. The surgical mask showed a reduction of 1.27 LOG_10_ (94.7%). For the community mask, a reduction of 0.82 LOG_10_ was observed (85.1%). The difference between the FFP-2 and the community mask was significant (*p*-value < 0.001). The surgical mask also showed a significantly higher reduction rate when compared to the community mask (*p*-value < 0.001). While the reduction rate of the FFP-2 mask was considerably larger than of the surgical mask, this effect was not significant.

The nanofleece materials showed high reduction rates when used as a double layer, with NFA2 showing a reduction of 1.34 LOG_10_ (95.5%) and NFB2 of 1.48 LOG_10_ (96.7%), respectively. There was no significant difference in the filtration efficiency of NFA2 and NFB2 when compared to the FFP-2 or the surgical mask. When compared to the community mask, a significantly increased filtration efficiency was only observed for NFB2 (*p* = 0.002), but not for NFA2. Both nanofleece materials showed reduced filtration efficiencies when used as a single layer (NFA and NFB). Both single-layer nanofleece materials showed significantly lower reduction rates than the FFP-2 mask. Thirty times washing (NFA2W) decreased the filtration efficiency of NFA2 from 95.5% to 92.5%. For NF2AW, a high variation in the measurements was observed with a standard error of 0.66 LOG_10_.

A high RH was present in compartment A with a mean of 83.6% (range: 78.6–90.2%). The RH measured in compartment B was lower with a mean RH of 66.5% and showed a higher variation (range: 40.8–76.1%). The mean temperature observed in compartment A was slightly lower with 21.8 °C (range: 20.9–22.7 °C) compared to a mean temperature of 23.7 °C (range: 23.4–24.9 °C) in compartment B.

Concerning the particle size of the viral aerosol, small particles with a diameter below 5 µm, dominated, representing 80.5% of total particles. In detail, the particle size percentages were as follows: 0.3–1 µm: 29.6%; 1–3 µm: 27.4%; 3–5 µm: 23.5%; 5–10 µm: 16.7%; 10–25 µm: 2.9%; >25 µm: 0.02%.

### 3.2. Structure of the Nanofleece Textiles after Aerosol Exposure

**Figure 4:**
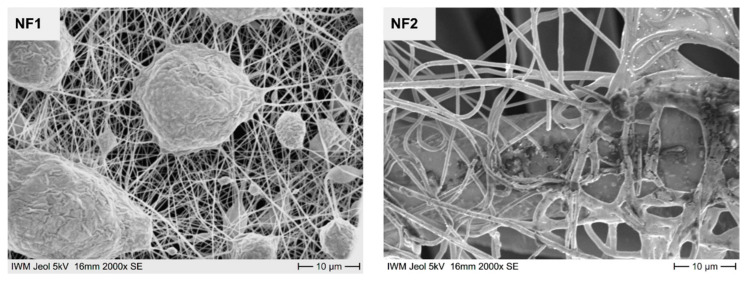
Microscopic images of NF1 and NF2 taken at 2000× magnification after aerosol exposure.

After aerosol exposure, microscropic images of NF1 and NF2 were taken and analyzed for structural changes (Figure 4). The microscopic images with 2000× magnification show that the superordinate fine mesh crosslinking of the electrospun nanofibrous web has not changed structurally. Even after aerosol exposure, the structure is intact. Furthermore, it cannot be seen that particles have clogged the mesh.

### 3.3. Antiviral Activity of Treated Textiles against SARS-CoV-2

It was ensured that all controls were valid as required by ISO 18,184: 2014. Neither a cytotoxic effect, an insufficient inactivation of antiviral activity after washing out the textiles, nor a weak cell culture sensitivity was observed in any untreated or treated textile sample (data not shown).

When screening the samples, three out of eight treated textiles, (C2, H2 and N2) showed significant activity against SARS-CoV-2 after an incubation time of 6 h. All other textiles showed only slight differences compared to the untreated textile (Table 3).

**Figure 5:**
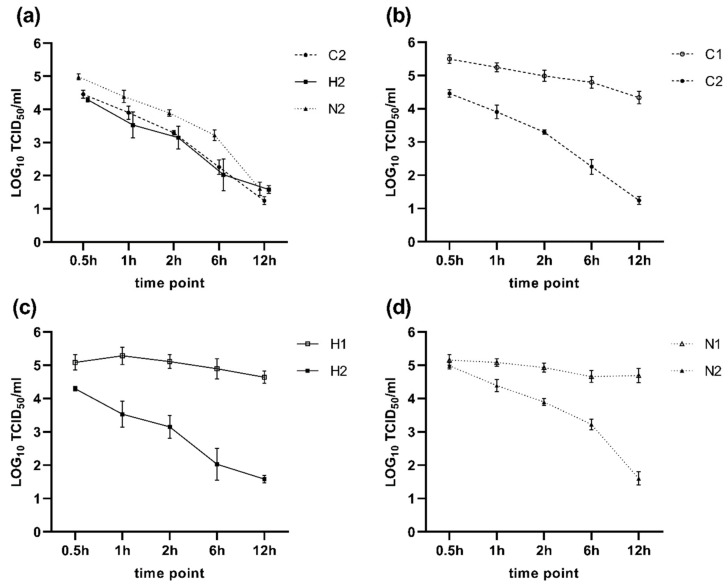
LOG_10_ SARS-CoV-2 concentration for the treated and untreated textiles at different points in time in TCID_50_/_mL_ washing out solution. The error bars indicate the 95% confidence intervals. Subfigure (**a**) shows the SARS-CoV-2 concentration for all treated textiles (C2, H2, N2). Subfigures (**b**–**d**) show the SARS-CoV-2 concentration of the untreated (1) and treated (2) textiles for each textile (C, H, N) separately.

Textiles C2, H2 and N2 were selected for further investigations. Results are summarized in Figure 5. All textile samples showed a disinfecting effect against SARS-CoV-2. In comparison to their untreated version, the textile samples induced a significant virus reduction at every investigated point in time (*p* < 0.001), except for textile N2 after 0.5 h of incubation (*p* = 0.067, Figure 5d).

After 12 h, all three textile samples showed a comparable virus reduction of about three LOG_10_ steps, which corresponds to a reduction of 99.9%. After six hours of incubation, Textile C2 and H2 achieved an average reduction of 2.5 (99.7%) and 2.8 LOG_10_ (99.8%) steps of virus titer reduction, respectively. Textile N2 showed an average reduction of 1.4 LOG_10_ (96%) steps at the same time point. Shorter incubation times showed less effect. However, after one hour, textile sample C2 achieved a mean virus titer reduction of 1.3 LOG_10_ (95%) compared to 1.8 LOG_10_ (98.4%) obtained by sample H2. Textile N2 showed a slower disinfection effect with a reduction of 0.7 LOG_10_ (80%) after one hour of incubation.

## 4. Discussion

Various recommendations to prevent transmission of SARS-CoV-2 within the community, such as physical distancing, personal hygiene and the general use of masks, exist worldwide [30]. Consequently, scientists have discussed the efficiency and hygienic aspects of face masks used in communities since the beginning of the pandemic [31,32]. Innovative textiles may have properties that are additionally useful to reduce the spread of SARS-CoV-2 in communities. Therefore, two approaches, which may also be used in combination, namely the use of nanofleece textiles as mask filter layers and antiviral treatment of the protective layers of masks, were evaluated in this study.

In the first part of the study, the filtration efficiency, which is a key element in mask testing [33] was evaluated for nanofleece textiles. It has been known for years that the particle filtration efficiency and viral filtration efficiency (VFE) of different face masks are not equivalent. Therefore, the VFE should be examined individually [34]. Even though there have been attempts to establish standard procedures concerning the investigation of the VFE in the past [35], to date no standard protocols exist [33]. Therefore, a new test system was developed in this study for the filtration experiments. Results from studies with different experimental setups should not be considered to be equivalent. Therefore, the inclusion of well-described reference mask materials is crucial when evaluating the filtration efficiency of newly tested mask materials [36]. In our study, three reference materials (FFP-2, surgical mask and community mask) were included. To date, only one other study, which was published recently, investigated the filtration efficiency of electrospun nanofilters regarding coronavirus aerosols. In this study, the Murine hepatitis virus A59 (MHV-A59) was used as a surrogate. A higher filtration efficiency of up to 99.9% regarding coronavirus aerosols was reported for electrospun materials [37].

The results of our study demonstrate that the two tested nanofleece textiles showed a reduction in FCoV in the aerosol that was almost on par with the reduction achieved by the FFP-2 mask material. However, this was only the case when the nanofleece textiles were used as a double layer. Konda et al. tested the filtration efficiency of different textiles in single and multiple layers and, accordingly, reported increased filtration rates for multiple layers of silk and chiffon [36]. We therefore recommend the use of double-layer nanofleece textiles. The larger standard deviation observed in the measurements with the nanofleece textiles compared to the reference materials may be explained by the random orientation of the fibers in the nonwoven mat, which may have led to differences in the mechanical filtration efficiency between individual textile samples [38].

Although we did not record the particle filtration efficiency during the VFE trials, we assume that the majority of the particles in the viral aerosol were in the respirable range because when determining the particle size distribution in the aerosol before, 80.5% of the particles had a size below 5 µm. The high VFE of >95% determined for the nanofleece materials indicates that small particles < 5 µm are also effectively filtered by nanofleece materials. We can also draw conclusions regarding the collected particle sizes because the collection cut-offs of the AGI-30 samplers are known. The lower cut-off is a particle diameter of 0.3 µm [39]. The upper cut-off is 15 µm; larger particles are not sampled in the collection fluid because they are collected at the tube wall by inertial force [40].

It was previously shown that nanofleece textiles retained their filtration capacities when cleaned in 75% ethanol, in contrast to meltblown textiles, which are used in FFP-2 masks [16]. It should also be considered that if appropriate decontamination methods, which maintain filter integrity, are applied, FFP-2 masks are not strictly for single use only. Fischer et al. also came to the conclusion that a decontamination with 70% ethanol led to a strong drop in the filtration efficiency of N95 respirators; however, other tested approaches including the treatment with dry heat, UV-C radiation or exposure to vaporized hydrogen peroxide allowed the reuse of respirators 1–3 times at comparable filtration efficiencies [41].

One of our hypotheses was that the nanofleece textiles would retain their filtration efficiency after washing. However, in our study, the filtration efficiency of NFA2 against FCoV decreased after 30 cycles of washing at 60 °C in a washing machine, and the experimental replicates with the washed material showed a high variation concerning the filtration efficiency. This leads to the conclusion that machine washing is not recommended for cleaning nanofleece masks, and that less stressful cleaning procedures such as handwashing should be used instead

The average RH in our experimental setup used for the VFE trials was very high, with 83.6% in compartment A and 66.5% in compartment B. Cherrie et al. (2019) measured the in-mask RH of people wearing respirators and reported a mean RH of 88.5%, which corresponds to compartment A in our experimental setup [42]. Therefore, the RH in our filtration trials was in a practically relevant range for evaluating face masks in terms of the protection of others, which ensures a good transferability of the results.

In our study, we aimed to determine the VFE against FCoV depending on the textile used. However, we did not consider the fitting of the different mask materials, which is important and usually performed as a standard testing method for face masks [33]. Instead, we used flat textiles for all trials, resulting in an ideal fit for all masks. In the study by Ueki et al., mannequin heads were used to take the effect of the mask fitting into account when determining the VFE of different mask types against SARS-CoV-2 [6]. They were able to show experimentally that an improved fitting leads to a higher VFE. Therefore, when producing masks using nanofleece textiles, it should be considered that the fitting should ideally be similar to FFP-2 masks in order to ensure a comparable VFE.

In the second part of this study, eight treatments of textiles were investigated concerning their antiviral activity against SARS-CoV-2. Specific treatments of textiles can give them antimicrobial properties and have recently become extremely important. However, the cleaning procedure of the masks is often complex and not clearly described [31]. Therefore, the textile treatments investigated in this study may contribute to the safer handling and wearing of community masks during the SARS-CoV-2 pandemic. All treatments were based on quaternary ammonium compounds. The antimicrobial effect is due to the interaction between the cationic ammonium group of QACs and the microbial cell membrane or the viral envelope, respectively, which is negatively charged. This results in a complex formation of microbes and surfactant and interrupts their protein activity [43]. It was described that the QAC concentration decreases through washing and boiling [44]. In this study, however, the antiviral effect was investigated after washing the fabrics ten times by hand and then autoclaving them. Therefore, it can be assumed that self-disinfection was still effective after hand washing ten times, since a sufficient concentration of QACs was still bound in the fabric.

Particular efforts should be made to avoid any negative health effects caused by treatment of the fabric when using treated textiles in face masks that are worn very close to the nose and mouth, over longer time periods, by many people worldwide. Therefore, treatments with heavy metal additives, which are commonly used as antimicrobials in textiles, were not included within this study. In this context, silver or copper are often used as an antimicrobial agent in textiles for clothing, and show significant antimicrobial effects [21]. A recent study tested the virucidal effect of cotton treated with silver and copper against feline coronavirus. The authors tested the effect after 2 h of contact with the virus and found a reduction of 3 LOG_10_ steps (TCID_50_) in silver-treated cotton and a reduction of 2.6 LOG_10_ steps (TCID_50_) in copper-treated cotton, which is slightly higher than in the present study at the same time point [45]. However, it was shown that silver is released from textiles [46] and leads to environmental exposure [47]. Data on antimicrobial coatings concerning ecotoxicological effects and effects on human beings are rare but should be urgently taken into consideration [48]. A very modern method is the impregnation of masks, such as surgical masks, with silver nanoparticles with the aim of self-disinfection, which the authors were able to successfully demonstrate in a study for various bacterial pathogens and also influenza virus after 15 min of exposure time. The authors also assume that with this modern method, fewer silver ions leak from the textiles and come into contact with the skin [49]. Further research should be conducted on this in the future.

The treatment of textiles that come into close contact to humans with biocidal active substances such as QACs has to be authorized by the European Chemicals Agency for the EU. In this study, three treatments of three different textiles (C2, H2, N2) from different companies showed a significant antiviral activity a few hours after virus inoculation. Presumably, the effect of the treatments is strongly dependent on the surface of the textile, its binding to the textile material and its interaction with other chemical finishes, such as hydrophobic finishes. Within the scope of this work, it can be shown that natural fibers such as cotton as well as synthetic fibers such as polyester can be treated with antiviral agents. The surface of the textiles can be specifically adjusted on the micro- and macro-scale level by changing the fiber geometry, the yarn configuration and the type of textile surface (weaving, knitting, nonwovens, etc.). This can change the effect of the treatments and requires further investigation.

The antiviral activity of the treated textiles was compared to the untreated textiles for each time point. This was to ensure that the observed effects were due to the treatment and not the textile itself. However, it is remarkable that one untreated textile (F1) also showed an antiviral activity after six hours of incubation (Table 3). This textile was made in India, where heavy use of pesticides in cotton planting is known [50]. This may explain the detected effect. In general, an infective virus titer reduction of two to three LOG_10_ steps (99–99.9%) is meant to be successful disinfection. Three LOG_10_ steps were achieved after 12 h of exposure in all further investigated textile samples. However, a reduction in infective virus of more than one LOG_10_ step, which means more than 90% virus inactivation, was obtained after a shorter time of 30 min for C2, after one hour for H2 and after two hours for N2. Earlier studies found virucidal activity against the enveloped vaccina virus and no effects on the non-enveloped poliovirus due to QAC-impregnated textiles [51]. In addition, other heavy metal-free treatments such as NaCl-salt coated fibers could be effective, since a deactivation of influenza virus was shown [52]. The efficiency of disinfection to prevent infection depends on the initial contamination with SARS-CoV-2. A minimum infective dose of slightly higher than 100 particles was assumed in a review literature article [53]; however, proof is still lacking. Viable SARS-CoV-2 was found in the saliva of symptomatic as well as asymptomatic patients [54]. Quantitative polymerase chain reaction revealed a viral load in saliva between 1.07–1.65 l LOG_10_ copies/mL and 5.58 LOG_10_ copies/mL in COVID-19 patients [55,56]. Droplets produced by the speaking, coughing and sneezing of SARS-CoV-2 positive persons contain more or fewer virus particles, probably depending on droplet size and individual virus load. The textiles absorb virus-containing droplets and, due to an effective textile treatment, virus particles are probably continuously inactivated over time. This improves the safety in the handling of contaminated masks and may prevent smear infections.

In conclusion, nanofleece textiles pose a promising alternative to FFP-2 masks concerning their filtration efficiency against coronaviruses, and antiviral textile treatments were proven to significantly decrease the contamination of mask materials with SARS-CoV-2. Because each potential transmission path of SARS-CoV-2, as well as the minimum infectious dose, are unknown to date, both approaches, which may also be used in combination, may be useful tools to lower the virus spread in communities.

## Figures and Tables

**Table 1:** Combinations of nonwoven textiles used for the FCoV filtration experiments.

Name	Layer 1	Layer 2	Layer 3	Layer 4	NaCl-Test (leakage %)
NFA	Spunbond (SBNF)	Electrospun (NF1)	Spunbond (SBNF)	-	28.1
NFB	Spunbond (SBNF)	Electrospun (NF2)	Spunbond (SBNF)	-	n.a.
NFA2	Spunbond (SBNF)	Electrospun (NF1)	Electrospun (NF1)	Spunbond (SBNF)	6
NFB2	Spunbond (SBNF)	Electrospun (NF2)	Electrospun (NF2)	Spunbond (SBNF)	n.a.
Surgical mask	Spunbond (SB25)	Meltblown (MB30)	Spunbond (SB25)		29.8
FFP-2 mask	Spunbond (SB25)	Meltblown (MB30)	Meltblown (MB30)	Spunbond (SB25)	11
Community mask	Canvas Woven	-	-	-	84.5

NF: nanofleece; MB: meltblown; SB: spunbond; n.a.: not analyzable.

**Table 2:** Summary of investigated textiles for their antiviral activity against SARS-CoV-2.

Textile ID	Textile Name	Textile Treatment	Applied on Textile in %
A1	Cotton Canvas 100	no treatment	
A2	Cotton Canvas 100	PolyHexaMethylene Biguanide (PHMB)	0.16
C1	Cotton Knit 180	no treatment	
C2	Cotton Knit 180	PolyHexaMethylene Biguanide (PHMB) proprietary binder	0.16 0.1
F1	Cotton Canvas 130	no treatment	
F2	Cotton Canvas 130	Dimethyloctadecyl [3-(trimethoxysilyl)propyl]ammonium chloride 68–70% Methanol 30–32%	1.22
F3	Cotton Canvas 130	Dimethyloctadecyl[3-(trimethoxysilyl)propyl]ammonium chloride 64–66% Methanol 34–36%	1.3
F4	Cotton Canvas 130	Dimethyloctadecyl[3-(trimethoxysilyl)propyl]ammonium chloride 38–42% Ethylene glycol 58–62%	2.1
H1	PES Canvas 120	no treatment	
H2	PES Canvas 120	Dimethyloctadecyl[3-(trimethoxysilyl)propyl]ammonium chloride 30–50% Methanol 0.1%–<1% Dimethylmyristylamine >1%–<5% N-Dimethyl-3-(trimethoxysilyl)propylamine >1%–<5%	2.0
M1	Cotton Canvas 90	no treatment	
M2	Cotton Canvas 90	(a.) Dimethyloctadecyl[3-(trimethoxysilyl)propyl]ammonium chloride 3–5% Methanol 0.5–1.7% *	8
		(b.) Polyetheramine-epichlorohydrin resin 8–21% Isotridecanol, branched, ethoxylated 2–10% Coco alkylbis(hydroxyethyl), ethoxylated, chlorides 5–6% Alcohols C9–11 ethoxylates 1.5–2.5%	2
N1	PES Canvas 120	no treatment	
N2	PES Canvas 120	See M2	See M2

* is removed in the textile application process—is not on the textile; PES: Polyester.

**Table 3:** Virus concentration and calculated antiviral activity of screened treated textiles in a two-fold approach (A, B).

Textile ID	TCID_50_/_mL_ Washing out Solution in log(10) after 6 h		Antiviral Activity in log(10) after 6 h	
	(A)	(B)	(A)	(B)
A1	3.85	4.35		
A2	4.1	4.6	−0.25	−0.25
C1	4.85	4.475		
C2	2.475	2.1	2.375	2.375
F1	3.725	3.6		
F2	2.35	2.35	1.375	1.25
F3	1.6	3.225	2.125	0.375
F4	1.85	2.35	1.875	1.25
H1	5.1	5.225		
H2	2.475	2.1	2.625	3.125
M1	4.725	4.85		
M2	2.975	3.225	1.75	1.625
N1	4.475	4.725		
N2	2.6	2.35	1.875	2.375

## Supplementary Materials

The following are available online at https://www.mdpi.com/article/10.3390/nano11082088/s1, Table S1: Characteristics of the nonwoven textiles, Table S2: Characteristics of the woven and knitted textiles, Table S3: CAS Numbers of substances used in treatments.
