# Supplementary material for: Innovative Textiles Used in Face Masks: Filtration Efficiency and Self-Disinfecting Properties against Coronaviruses
Source: Nanomaterials (Basel). 2021 Aug 17;11(8):2088. doi: 10.3390/nano11082088 (PMC8402066; doi:10.3390/nano11082088)
Supplement: Supplementary file 1 [file nanomaterials-11-02088-s001.zip › nanomaterials-1313398-supplementary.pdf]

Supplementary Materials

Innovative Textiles Used in Face Masks: Filtration Efficiency and Self-Disinfecting Properties against Coronaviruses

Paul Siller [1], Janina Reissner [1], Sabrina Hansen [1], Michael Kühl [1], Alexander Bartel [2], David Schmelzeisen [3], Thomas Gries [3], Uwe Roesler [1] and Anika Friese [1,*]

Table S1. Characteristics of the nonwoven textiles.

Name	Polymer	Production Method	Specific Weight [g/m²]	Thickness [mm]	Air Permeability. Pressure Drop [Pa] at 95 l/min	Air Permeability. Pressure Drop [Pa] at 160 l/min
NF1	PVDF	electrospinning	6,27	0.029	301.6	515.7
NF2	PVDF	electrospinning	2,52	*	95	158.5
MB30	PP	meltblown	29.29	0.285	862.6	1466.0
SB25	PP	spunbond	25.33	0.255	19.4	36.8
SBNF	PP	spunbond	27.82	0.224	38.6	71.0

NF: nanofleece; MB: meltblown; SB: spunbond; PVDF: Polyvinylidenfluorid; PP: Polypropylen; *below measurement limit.

Table S2. Characteristics of the woven and knitted textiles.

	Name	Type	Material	Specific weight [g/m²]	Thicknes s [mm]	Yarn Count			Mesh Count		
						Warp threads /cm	Weft threads /cm	Threads /cm²	MS/cm	MR/cm	Meshes/ cm²
A1	Cotton Canvas 100	Woven Canvas	Cotton	101.04	0,2	59	42	2450			
A2	Cotton Canvas 100	Woven Canvas	Cotton	101.19	0,205			2348			
C1	Cotton Knit 180	RL Knit	Cotton	187.24	0,671				14	20	272
C2	Cotton Knit 180	RL Knit	Cotton	172.87	0,607				14	19	264
F1	Cotton Canvas 130	Woven Canvas	Cotton	130.91	0,326	55	29	1584			
F2	Cotton Canvas 130	Woven Canvas	Cotton	129.04	0,315	54	28	1539			
F3	Cotton Canvas 130	Woven Canvas	Cotton	131.99	0,312	55	27	1511			
F4	Cotton Canvas 130	Woven Canvas	Cotton	124.75	0,284	55	28	1546			
H1	PES Canvas 120	Woven Canvas	Polyester 97%. Elasthan 3%	122.45	0,275	56	28	1535			
H2	PES Canvas 120	Woven Canvas	Polyester 97%. Elasthan 3%	125.67	0,277	55	29	1571			
M1	Cotton Canvas 120	Woven Canvas	Cotton	122.31	0,372	42	32	1329			
M2	Cotton Canvas 120	Woven Canvas	Cotton	122.31	0,372	42	32	1329			
N1	PES Canvas 90	Woven Canvas	Polyeste r	92.11	0,245	41	29	1203			
N2	PES Canvas 90	Woven Canvas	Polyeste r	92.11	0,245	41	29	1203			

Table S3. CAS Numbers of substances used in treatments.

**Substance**	**CAS #**
Dimethyloctadecyl[3-(trimethoxysilyl)propyl]ammonium chloride (F2, F3, F4, M2, N2)	CAS 27668-52-6
PolyHexaMethylene Biguanide	CAS 1802181-67-4
Ethylene glycol	CAS 107-21-1
Dimethyloctadecyl[3-(trimethoxysilyl)propyl]ammonium chloride (H2)	CAS 41591-87-1
Methanol	CAS 67-56-1
Dimethylmyristylamine	CAS 112-75-4
N.N-Dimethyl-3-(trimethoxysilyl)propylamine	CAS 2530-86-1
Isotridecanol, branched, ethoxylated	CAS 69011-36-5
coco alkylbis(hydroxyethyl),ethoxylated, chlorides	CAS 61791-10-4
Alcohols C9-11 ethoxylates	CAS 68439-46-3
